# Flow Experiences in Shopping Activities: Testing Materialistic Goal Orientation as an Antecedent

**DOI:** 10.1177/00332941231159615

**Published:** 2023-02-23

**Authors:** Amy Isham, Tim Jackson

**Affiliations:** School of Psychology, 7759 Swansea University, Swansea, UK; Centre for the Understanding of Sustainable Prosperity (CUSP), 3660 University of Surrey, Guildford, UK

**Keywords:** flow, materialistic goal orientation, shopping, store attitudes, purchase intentions

## Abstract

Given that flow experiences when shopping can encourage positive brand attitudes and purchase behaviours, consumer psychologists are interested in the antecedents to flow within retail environments. Emerging findings suggest that a materialistic goal orientation can undermine an individual’s tendency to have optimal experiences of flow. However, this existing work has been conducted largely within the field of Environmental Psychology and thus focused on flow experiences that occur in more ecologically sustainable activities. We hypothesized that materialism may not have the same flow-limiting effects when participants are engaged in shopping activities, which are more in line with the goals of highly materialistic individuals. Across two studies, we tested the relationship between materialism and the experience of flow during shopping activities using cross-sectional (*N* = 886) and experimental (*N* = 140) methods. Contrary to our hypothesis, both studies documented a negative effect of materialism on flow experiences when shopping, and this was not moderated by the type of store browsed. Accordingly, it appears that a materialistic goal orientation limits the extent to which people can have enjoyable flow experiences even during activities which are consistent with the life goals of highly materialistic individuals. We discuss the implications of these findings for wellbeing, marketing, and sustainability.

## Introduction

This research explores the relationship between a materialistic goal orientation and the experience of a psychological state known as ‘flow’ when shopping. Flow experiences occur when individuals are “so involved in an activity that nothing else seems to matter” (Csikszentmihalyi, 1992, p. 4). Such experiences have been shown to be highly enjoyable and an important component of individual wellbeing (Isham & Jackson, 2022). The field of Consumer Psychology has developed an interest in flow following research showing that flow experiences when shopping can influence consumer experiences and behaviour (Richard & Chebat, 2016). In line with this, studies have begun to look at how to design retail environments to support flow experiences (Martins et al., 2019; Sarkar & Khare, 2019).

At the same time, the field of Environmental Psychology has started to explore how flow experiences can act as a tool to support sustainable outcomes such as engagement in activities with lower greenhouse gas emissions (Isham et al., 2019; Kim & Thapa, 2018). Part of this work has focused on the relationship between materialistic goal orientation and the experience of flow. Studies have documented that individuals with a stronger materialistic goal orientation tend to be less inclined to experience flow (Isham et al., 2021a). In some cases, it has been demonstrated that experimentally increasing the salience of materialistic goals can directly undermine the experience of flow (Isham et al., 2021b). Given the focus on promoting flow in more environmentally friendly activities, this field of research has tended to examine flow in contexts that do not necessarily incur high environmental costs such as artwork or meditation. Flow experiences that occur in more consumption-based activities such as shopping have not been granted proper attention in the Environmental Psychology literature.

Consumer psychologists are interested in locating the determinants of flow experiences when shopping. Whilst work within Environmental Psychology suggests that strong materialistic goals can impede flow experiences, this research has not considered flow experiences that occur within the shopping context. In a shopping environment, materialistic goals may not hold the same flow-limiting effects given that shopping is an activity that is consistent with the desires, likes, and interests of those individuals with stronger materialistic goals (Sirgy et al., 2020). In fact, materialism may even *promote* flow in a shopping context given that greater liking and perceived importance of an activity have been shown to be precursors to high levels of intrinsic motivation (Engeser & Baumann, 2016), an important component of flow. In this research we therefore set out to test whether materialistic goals also undermine flow experiences in shopping activities, or not. To this end, we conducted two studies to examine the nature of the relationship between materialistic goals and flow experiences when shopping using both cross-sectional (Study 1) and experimental (Study 2) methods.

### Flow

Flow describes a state whereby an individual is totally immersed in an optimally challenging activity. This is to the extent that they lose track of time and may forget about everyday concerns (Csikszentmihalyi, 1992). An individual ‘in flow’ is completely concentrated, feels in control, has clear goals, and receives continual feedback concerning their progress towards these goals. Their actions may also feel effortless as they are not aware of any conscious effort to initiate them. The flow state is said to be enjoyable and intrinsically rewarding. Thus, once an individual has experienced flow in an activity, they should be motivated to experience it again (Csikszentmihalyi, 1992). Alongside being inherently enjoyable, flow experiences have been shown to have many benefits for personal wellbeing including more positive emotions (Rogatko, 2009), greater life satisfaction (Tse et al., 2021) and stronger feelings of fulfilment (Asakawa, 2004).

### Flow in Consumer Psychology

Consumer psychologists recognise flow as an important factor that influences consumer experiences and behaviour (Richard & Chebat, 2016). It has been suggested that shopping is an activity that can support flow experiences (Wang & Hsiao, 2012) because it can provide challenges (e.g., budgeting, choosing between products), offer clear goals, and be fun and enjoyable. Research has documented that consumers are often motivated to visit shopping malls to experience flow (Ahmed et al., 2007). When recounting flow experiences during shopping, some US university staff and students described flow as a “shopping high” (Wang & Hsiao, 2012).

Part of the reason why flow has generated so much interest in the field of Consumer Psychology is the number of positive commercial outcomes it has been associated with. For example, the experience of flow on an e-commerce site has been linked to a greater subsequent desire to acquire more information about a product, brand, or service (Korzaan, 2003), greater intention to revisit the site (Skadberg & Kimmel, 2004) and spending more time on it (Ettis, 2017). As well as influencing consumer browsing behaviour, flow has been linked to purchase outcomes. Experiencing flow on an e-commerce site is associated with more purchases made, whether these are planned (Niu & Chang, 2014) or unplanned/impulsive (Hsu et al., 2012). Flow can enhance future shopping and purchase intentions (Gao & Bai, 2014; Ham et al., 2016) with the store/brand. The experience of flow when shopping has also been linked to greater e-store loyalty (Hsu et al., 2013; O'Cass & Carlson, 2010), higher levels of brand identification (Lin et al., 2019) and more positive attitudes towards the e-store and associated brands (Mathwick & Rigdon, 2004).

Given the proposed commercial benefits of flow experiences, research has begun to examine the antecedents of flow in retail environments. Much of this research has focused on the design of retail environments or situational characteristics of the specific shopping task. For example, perceived e-store ease of use, credibility, and interactivity have been shown to promote flow experiences (Hsu et al., 2013; Martins et al., 2019; Novak et al., 2000; Sarkar & Khare, 2019; Wu & Chang, 2005). Further, flow has been suggested to be most likely to occur when shoppers have a task-specific focus, rather than just browsing for fun (Rettie, 2001). Different types of shopping may also offset the balance of challenge/skill and, in turn, influence the likelihood of flow occurring. When conducting interviews with university staff and students, Wang and Hsiao (2012) reported that interviewees noted that shopping for a gift for others was more challenging than shopping for themselves because they often did not have a clear idea of the criteria to use to make the best choice. Another participant noted that picking up prescriptions was boring, perhaps because the activity requires very little skill. Less work has explored the individual difference factors that could influence flow experiences when shopping. Whilst Smith and Sivakumar (2004) proposed that consumer characteristics such as self-confidence and perceived risk might influence flow experiences when shopping, the influence of consumers’ materialistic goal orientation has not previously been considered in this context.

### Materialistic Goal Orientation

Materialism, in a broad sense, is commonly defined as “individual differences in people’s long-term endorsement of values, goals, and associated beliefs that center on the importance of acquiring money and possessions that convey status.” (Dittmar et al., 2014, p. 879). Historically, research has tended to consider materialism as either a personality trait, value, or goal orientation (Kasser, 2018). As a system of personality traits, materialism is suggested to be composed of the traits of envy (a disliking of someone because they own a possession that one desires), possessiveness (a need to have sole ownership of items along with great worry about their loss), and non-generosity (an objection to donate possessions to others or share) (Belk, 1985; Ger & Belk, 1996). When considered as a value (Richins, 1994), materialism reflects the belief that acquiring money and material possessions is highly important, and will lead to higher levels of happiness, success, and status for the individual. Using this approach, which is particularly common in the field of Consumer Research, materialism is often measured using the Material Values Scale (MVS) which includes items such as “I like a lot of luxury in my life” and “I’d be happier if I could afford to buy more things” (Richins & Dawson, 1992).

In this research we consider materialism as a goal orientation (Kasser & Ryan, 1996). Here, materialism reflects the relative importance placed on goals such as money, possessions, and image (often known as extrinsic goals) in comparison to goals such as health, relationships, and personal growth (often known as intrinsic goals). Using the goal orientation approach, which is particularly common in psychological research (Dittmar & Hurst, 2018), materialism if often measured using the Aspiration Index (AI) (Kasser & Ryan, 1996). This index asks individuals to rate how important different goals (e.g., “to be rich” and “to grow and learn new things”) are to them and considers a greater relative importance placed on extrinsic goals to indicate a materialistic goal orientation. We chose to consider materialism as a goal orientation and measure it using the Aspiration Index because such an approach best reflects the understanding that materialism exists within a broader context of other values and life goals (Burroughs & Rindfleisch, 2002). The AI allows for examination of how people prioritise materialistic relative to other life goals whilst the MVS only measures materialistic values in isolation (Parker & Ivtzan, 2016).

### Materialistic Goal Orientation and Flow in Environmental Psychology

Emerging findings within the field of Environmental Psychology are starting to suggest that flow experiences can be used to promote sustainable outcomes. For example, Isham et al. (2019) highlighted that flow experiences tended to be more likely to occur in activities with low environmental costs, such as sports, arts, and contemplative practices, and therefore represented a means of improving wellbeing in more environmentally friendly ways. Tourists’ experiences of flow during a trip to South Korea has been positively linked to their engagement in pro-environmental behaviours whilst at their destination (Kim & Thapa, 2018). Emerging findings are also starting to document an increase in self-transcendent values (which prioritise the wellbeing of other people and the environment) as people come to experience flow more frequently (Isham & Jackson, 2023).

Following a large body of research documenting that materialistic goal orientation is related to lower levels of various aspects of individual wellbeing including life satisfaction and sense of purpose in life (Dittmar et al., 2014; Dittmar & Isham, 2022; Kasser, 2018; Moldes & Ku, 2020), research has sought to determine whether materialistic goals are also linked to less frequent experiences of flow. Materialistic goals are encouraged by consumer capitalism, which remains the dominant economic framework in many developed nations (Kasser et al., 2007). Their consequences are important to study because they inform us of how our economic systems impact upon citizens’ quality of life. Work conducted so far has documented a negative association between materialism and flow experiences (Isham & Jackson, 2022). Results from Isham et al. (2021a; 2021b) and Isham et al. (2022) demonstrated, using cross-sectional surveys, that those individuals reporting stronger materialistic goals and values also tended to be less flow prone. That is, materialistic individuals are less likely to experience the characteristics of flow in their day-to-day lives. Further, Isham et al. (2021a) reported that temporarily increasing the salience of materialistic goals through experimental priming led individuals to report significantly poorer quality flow experiences in a subsequent activity, in comparison to a control group who had not had the salience of their materialistic goals heightened. These experimental studies suggest that the presence of strong materialistic goals can directly limit an individual’s ability to experience flow.

This previous work was grounded in a framework whereby flow was considered as a way of enhancing both psychological and ecological wellbeing. As such, the activities chosen to provide the opportunity for flow in these experimental studies (Isham et al., 2021a) mainly included activities such as artwork, mindfulness, and puzzle games, which previous research had shown to be associated with less materialistic behaviours and values.

### Materialistic Goal Orientation and Flow Experiences When Shopping

Consumer psychologists want to determine the antecedents to flow experiences within shopping environments to promote positive brand attitudes and purchase behaviours. The field of Environmental Psychology suggests that strong materialistic goals may impede an individual’s likelihood of experiencing flow. Whether or not the negative effect of materialistic goals on flow experiences extends to shopping contexts has important practical implications. Retail environments often encourage materialistic goals, for example through the presence of consumer advertisements. However, if materialistic goals undermine flow experiences, which are suggested to promote purchase behaviours, this means that marketers and retailers may undermine the very commercial outcomes that they seek to produce. Further, if sustainability practitioners perceive reducing the prevalence of materialistic goals to be a means of encouraging flow experiences in sustainable activities, then this strategy could backfire if reducing materialistic goals encourages flow experiences, and thus purchase behaviours, in shopping contexts.

To inform the marketing/retail and sustainability applications of flow it is important that we examine whether strong materialistic goals have the same or a different impact on the experience of flow in shopping activities, as they do on flow experiences in more sustainable activities. Prima facie, it would seem wrong to assume that individuals with stronger materialistic goals will still report poorer quality flow experiences than those individuals with weaker materialistic goals when the flow-inducing activity itself is more in line with the interests and goals of highly materialistic individuals.

### Theory Development

Whilst the negative effect of materialistic goals on flow experiences has been demonstrated in previous work, studies are only beginning to try to understand why strong materialistic goals may have their flow-limiting effects. Isham et al. (2021b) explored the potential role of self-regulation – which refers to each individual’s ability to manage their own thoughts, emotions and behaviours – in explaining this negative relationship. They found that more materialistic individuals tended to have lower trait levels of self-regulatory strength which, in turn, was linked to a lesser tendency to experience flow in their daily lives. Low levels of self-regulatory strength may limit the extent to which individuals can effectively manage their attention or develop sufficient skills to experience flow. What’s more, the way in which individuals use their self-regulatory strength also seems to be relevant for understanding why materialistic values and goals are negatively associated with flow experiences. Isham et al. (2021b) found that highly materialistic individuals tended to try to avoid being in contact with negative thoughts, feelings, and events. The avoidance of negative states appeared to be negatively related with the tendency to experience flow, and this effect was partly explained by the fact that trying to avoid negative states was linked to lower levels of self-regulatory strength. So far, these relationships have only been demonstrated at a cross-sectional level, and thus, it is not clear to what extent the outlined mechanisms have causal roles to play in explaining why materialistic goals have been documented to undermine flow experiences in experimental research.

We suggest that another reason why materialistic goals undermine flow experiences can be rooted in Self-Determination Theory (SDT, Deci & Ryan, 2000). SDT provides a meta-theory for the study of human motivation (Ryan & Deci, 2002). One of the core contributions of SDT has been the distinction between different types of motivation. At a broad level, there is the distinction between intrinsic and extrinsic motivation. Intrinsic motivation involves engaging in an action because the action itself in enjoyable, interesting, and rewarding. This type of motivation can be considered as autonomous. In contrast, extrinsic motivation involves engaging in an action to obtain a separate contingency. The final goal is not engagement in the activity, but rather the avoidance of punishment or obtainment of a reward. Extrinsic motivation can be considered as a controlled form of motivation because the achievement of the contingencies is usually controlled by another external agent (Deci & Ryan, 2012). The founders of SDT, Deci & Ryan (2000), already noted the commonalities between flow theory and SDT, highlighting that flow was “the prototype of intrinsically motivated activity.” (pp. 260). Flow has continued to be described as a state of intrinsic motivation (Baumann & Scheffer, 2001; Kehr, 2004; Swiatczak, 2021) and empirical studies have documented that people experience more intense episodes of flow in activities that are intrinsically motivated (Kowal & Fortier, 1999; Mills & Fullagar, 2008).

Motivation concerns *why* people pursue different actions, whereas the contents of a goal describe *what* people are pursuing. Similarly to the distinction between different types of motivation, Deci and Ryan (2000) noted that goal contents can themselves be considered as intrinsic or extrinsic. Drawing on Kasser and Ryan’s (1996) work, intrinsic goal contents involve personal growth, quality relationships, and community involvement. Intrinsic goal contents are inherently rewarding to pursue because they help to satisfy psychological needs such as relatedness and competence. In contrast, extrinsic goal contents involve money, fame, and image. Such goals are less well equipped to directly satisfy psychological needs (Sheldon et al., 2004) and are usually pursued as a means of achieving external contingencies such as higher feelings of self-worth (Gunnell et al., 2014). We have already noted how materialistic goals are extrinsic (Kasser & Ryan, 1996).

It has been suggested that the pursuit of extrinsic goals is more likely to be extrinsically motivated whilst intrinsic goals are more likely to be pursued for intrinsic reasons (Carver & Baird, 1998). Studies have supported this positive association between the ‘what’ and ‘why’ of behaviour (Conti, 2000; Ingledew et al., 2009). If individuals holding extrinsic goals are more likely to be extrinsically, rather than intrinsically motivated, then this may mean that such individuals are less likely to experience flow, given that flow is an experience of intrinsic motivation. This could explain why individuals with strong materialistic goal orientation are less inclined to experience flow.

However, whilst goal contents and motivations may be positively associated, they are still distinct concepts (Sheldon et al., 2004). This means that extrinsic goals could in principle also be intrinsically motivated. The shopping context may present an example of when such a case occurs for individuals with materialistic goal orientation. Shopping is an activity that is largely in line with the goal contents of more materialistic individuals and several studies have documented that people with strong materialistic goals have greater liking for shopping (Fitzmaurice & Comegys, 2006; Goldsmith et al., 2011). We may therefore expect them to be more intrinsically motivated to engage in shopping activities. In other words, they may have a materialistic (extrinsic) goal contents (e.g., I shop to improve my image) but be intrinsically motivated (e.g., because I enjoy looking for products that will make me look good). Further work has shown that perceived outcome importance is positively related to flow experiences in an activity (Engeser & Baumann, 2016) and that the meaningfulness of an activity, defined as “the value of the task goal or purpose, judged in relation to the individual’s own ideals or standards” (p. 774), is a precursor to high levels of intrinsic motivation. Accordingly, due to the greater liking and perceived importance that individuals with materialistic goal orientation grant to shopping activities, we may expect them to be *more* likely to experience flow in such activities.

The hypothesized positive affect of materialistic goals on flow experiences when shopping may be moderated by the type of shopping that an individual is engaged in. Certain types of shopping are more strongly aligned with materialistic goals and hence may prompt higher levels of engagement and intrinsic motivation. For example, ‘conspicuous consumption’ refers to the acquisition of a product or service driven not by the functional features/use of that product, but rather by the desire to display ownership of it and exhibit a certain wealth, status, or social standing (O’Cass & McEwen, 2004; Veblen, 1899). Goods purchased through conspicuous consumption are often expensive, fashionable, or scarce. Numerous studies have shown that materialistic goals are associated with a preference for conspicuous consumption (Chacko et al., 2018; Podoshen & Andrzejewski, 2012). One example of a materialistic goal is ‘image’ (Kasser & Ryan, 1996). Those scoring higher on measures of materialism therefore tend to place greater value on items that can be worn or seen in public (Richins, 1994) and prestigious brands (Zarco, 2014). If stronger materialistic goals are associated with greater preference for conspicuous consumption, then we might expect that materialistic goals would have a stronger positive affect on flow experiences in shopping activities that involve ‘conspicuous’ luxury items, in comparison to when shopping for purely functional goods that are not as visible and hence do not have the same image-enhancing properties.

### The Present Research

The present research aimed to examine the influence of materialistic goal orientation on the experience flow when shopping. To this end, two studies were conducted. Study 1 starts by testing the relationship between materialistic goal orientation and the tendency to experience flow when shopping cross-sectionally. This allows us to determine whether, at the trait level, those individuals with a stronger materialistic goal orientation are more or less likely to experience flow when shopping. Study 2 then moves on to explore how the relationship operates causally, this time at the state level. By experimentally priming materialistic goals to be more salient, we could determine if a materialistic goal orientation directly causes differences in flow experiences when shopping. Study 2 also allowed for the testing of a moderation effect to see if the type of shopping (conspicuous versus inconspicuous goods) impacted the effect of materialistic goal orientation on flow experiences.

As a secondary aim of this research, we also sought to determine whether we could replicate the finding that experiences of flow when shopping lead to more positive brand attitudes and purchase intentions. Flow is of interest to consumer psychologists largely because of the hypothesis that it leads to positive commercial outcomes (e.g., Gao & Bai, 2014; Ham et al., 2016). However, one problem we have noticed with the study of flow within Consumer Psychology is that the concept is not always consistently defined and measured across studies. Whilst Csikszentmihalyi (1992) outlined nine characteristics of flow, each of which are assessed in the most standard measures of flow such as the Flow State Scale 2 (Jackson & Eklund, 2002), the same nine characteristics have not always been used in Consumer Psychology research. Sometimes flow is considered as high levels of concentration along with an optimal matching of challenge and skill (Hoffman & Novak, 1996). At other times it is simply a combination of enjoyment and concentration (Ettis, 2017) or enjoyment and losing track of time (Skadberg & Kimmel, 2004). By using a measure of flow experience that includes all nine of the proposed components of flow we hoped to gain a more comprehensive examination of how flow experiences are related to subsequent attitudes and behavioural intentions towards an e-store.

The outcomes of this research make several important contributions to academic understandings and have practical implications for studies of wellbeing, for marketers, and for sustainability practitioners alike. Theoretically, they strengthen the evidence base surrounding the consequences of materialistic goal orientation on an important aspect of wellbeing and provide novel insights concerning the extent to which such consequences may be activity-specific, or not. More practically, the findings can inform marketers about the efficacy of materialistic messaging in advertisements and shopping environments for encouraging flow during the shopping experience. In addition, they inform sustainability practitioners about the extent to which trying to reduce materialistic values may, or may not, produce unintended effects by enhancing flow (and thus, potentially promoting further engagement) in consumption-based activities.

In line with the literature reviewed and the proposed theoretical framework, the following hypotheses were made. H1 was examined in Study 1. H2 – H4 were examined in Study 2.

H1 Materialistic goals will be positively associated with the tendency to experience flow when shopping.

H2 Participants exposed to a materialistic prime will report higher quality of flow experiences in a subsequent shopping activity, in comparison to a control group who are not exposed to the materialistic prime.

H3 The effect of a materialistic prime on subsequent flow experiences in a shopping activity will be moderated by the type of e-store browsed. This moderation effect will operate such that exposure to a materialistic prime will have a stronger positive effect on flow experiences when shopping for luxurious, expensive, conspicuous items, in comparison to when shopping for functional, non-conspicuous items.

H4 Higher quality experiences of flow when shopping will be associated with more positive attitudes and behavioural intentions towards the store browsed.

## Study 1

Study 1 used an online survey to test whether, at the trait level, holding a stronger materialistic goal orientation is significantly linked to the tendency to experience flow in shopping activities. In contrast to existing findings which report a negative correlation between materialistic goals and the tendency to experience flow in either general day-to-day life or more ecologically sustainable activities (Isham et al., 2021a; 2021b; Isham et al., 2022), we hypothesized that materialistic goals would be positively associated with the tendency to experience flow when shopping (H1). This is because we expected that shopping is an activity that is more enjoyable, interesting, and important to individuals with a materialistic goal orientation. Accordingly, they should be more intrinsically motivated to engage in shopping activities, enjoy them more, and be more concentrated on them, which are all core characteristics of the flow state.

### Materials and Methods

#### Participants

A sample of 886 adults in the United Kingdom was recruited by an external market research company. Attempts were made to recruit individuals spanning across different age groups, socioeconomic status, geographical regions, educational attainments, and employment status. In terms of gender, 396 respondents were female and 490 were male. The median and mean age group were both 45–54 years old (min = 18–24, max = 65+). The median level of education completed was A-levels/college diploma. A total of 520 respondents were employed, 8 were students and 347 were unemployed/retired (11 did not specify their highest level of education). The majority (95%) identified their nationality as British. The study passed an ethical assessment in line with the University’s ethical guidelines and informed consent was gained for all individuals before they entered the online survey.

#### Measures

##### Materialistic Goals

Materialistic goals were measured using the 14-item shortened version of Kasser and Ryan (1996) Aspiration Index (Martos et al., 2006). This scale assessed how important respondents deemed different life goals. Some of these goals are classified as extrinsic (e.g., “to be rich” and “to achieve the ‘look’ I’ve been after”) whilst others are intrinsic (e.g., “to know ad accept who I really am” and “to help others improve their lives”). Respondents rated how important each goal was to them on a scale of 1 (*not at all*) to 7 (*very*). The internal reliability of both the extrinsic (α = .87) and intrinsic (α = .89) items was good. Materialism is considered as placing a greater emphasis on extrinsic relative to intrinsic goals. Therefore, the mean score on the intrinsic items was subtracted from the mean score on the extrinsic items, yielding a Relative Extrinsic–Intrinsic Goal Orientation (REIGO) score which reflects the relative importance placed on extrinsic versus intrinsic goals.

##### Flow Proneness

Participants’ tendency to experience flow in shopping activities was assessed using the Short Dispositional Flow Scale 2 (S-DFS2; Jackson et al., 2008) which is a nine-item scale tapping into all Csikszentmihalyi’s proposed components of flow. These are challenge-skill balance, action-awareness merging, clear goals, unambiguous feedback, concentration on the task at hand, sense of control, loss of self-consciousness, time transformation, and intrinsically motivating. Participants rated, on a scale of 1 (*never*) to 5 (*always*), how often they experienced each characteristic, in general, when engaged in shopping activities. Example items included “I feel I am competent enough to meet the demands of the situation” and “I am completely focused on the task at hand”. This scale showed good reliability in the present study (α = .89).

#### Analysis Plan

The relationships between demographic variables and both materialistic goals and flow proneness in shopping were first tested using Pearson correlations and independent samples t-tests. This was to determine if the demographic variables should be added as controls within the analysis. Multicollinearity tests were used to determine whether it was appropriate to conduct a regression analysis to assess the amount of variance in flow proneness in shopping was accounted for by materialistic goals. Multicollinearity was considered to not be a problem if the variance inflation factors (VIF) were less than 4.0, and the tolerance greater than 0.2 (Hair et al., 2010). A hierarchical linear regression was employed to determine how much additional variance materialistic goals accounted for on top of the demographic controls.

### Results and Discussion

Initial correlations (see Supplementary Materials) demonstrated significant associations between the demographic variables, materialistic goals, and flow proneness for shopping. Accordingly, demographic factors were controlled for in the regression analysis. VIF and tolerance values were all less than 1.6 and greater than .65, respectively, indicating that we did not have a serious problem with multicollinearity. A hierarchical linear regression analysis (see Table 1) that controlled for age, gender, socioeconomic status, educational attainment, and employment status highlighted that the addition of the materialistic goal orientation variable led to a significant improvement in the R^2^ value for the model, R^2^ change = .024, *p* < .001. Materialistic goal orientation displayed a significant and negative association with flow proneness for shopping. This finding is in the opposite direction to that which we hypothesized, but consistent with previous research on non-shopping activities (Isham et al., 2021a; 2021b; Isham et al., 2022). It therefore appears that the negative impact of materialistic goals on flow experiences does extend to shopping-based activities, even though the size of this negative effect is small accounting for 2% of variation in flow proneness scores. This suggests that engagement in a goal-consistent activity does not protect against the negative effects of materialistic goals on flow.

**Table 1.** Hierarchical Linear Regression Assessing the Effect of the Strength of Materialistic Goal Orientation on Flow Proneness in Shopping.

	*t*	*p*	β	95% CI	*F*	*df*	*p*	Adj. *R*^2^	*R*^2^ Change
**Step 1**					4.58	5, 865	<0.001	0.02	
Age	3.76	0.01	0.11	0.14, 0.79					
Gender (0 = male, 1 = female)	3.22	<0.001	0.11	0.53, 2.20					
Socioeconomic status	−1.11	0.27	−0.05	−0.31, 0.09					
Educational attainment	.81	0.42	0.03	−0.24, 0.59					
Employment status (0 = unemployed, 1 = employed)	1.47	0.14	0.06	−0.23, 1.60					
**Step 2**					7.50	6, 864	<0.001	0.04	0.024
Age	1.41	0.16	0.06	−0.10, 0.58					
Gender (0 = male, 1 = female)	2.52	0.01	0.09	0.24, 1.91					
Socioeconomic status	−1.28	0.20	−0.05	−0.32, 0.07					
Educational attainment	0.25	0.80	0.01	−0.36, 0.47					
Employment status (0 = unemployed, 1 = employed)	1.80	0.07	0.07	−0.08, 1.74					
Materialistic goal orientation	−4.64	<0.001	−0.17	−1.03, −0.42					

## Study 2

Study 2 employed an experimental design to test whether priming a heightened salience of materialistic goals impacts upon people’s flow experiences in a subsequent shopping activity. Our original hypothesis outlined in the introduction was that priming stronger materialistic goal orientation would lead participants to report higher quality flow experiences in the subsequent shopping activity (H2). However, now that Study 1 had documented a negative association between materialistic goals and the tendency to experience flow when shopping, we were less confident that a positive association would emerge in this experimental study. Study 2 also tested the moderation effect of the type of shopping engaged in. We hypothesised that materialistic goals would have a stronger positive effect on flow experiences when shopping for luxurious, expensive, conspicuous items, in comparison to when shopping for functional, non-conspicuous items (H3). Further, Study 2 aimed to document the positive effect of flow experiences when shopping on consumer attitudes and behavioural intentions found in previous research, but this time using a more encompassing measure of flow. We hypothesized that higher quality experiences of flow when shopping will be associated with more positive attitudes and behavioural intentions towards the store browsed (H4).

### Materials and Methods

#### Participants

A total of 140 students, recruited as part of the UK-based University’s research participation system, took part in the study. Of these 140 students, 18 (13%) were male and 122 (87%) were female. Participants mean age was 19.35 (*SD* = 1.38). The majority (74%) were British. All participants received one lab token for their participation in the study. The study passed an ethical assessment in line with the University’s ethical guidelines and informed consent was gained for all individuals before they entered the online survey.

#### Design

The study employed a between-participants design whereby the first independent variable was whether participants were in the materialism or control prime group. The salience of materialistic goals was manipulated using a ‘scrambled sentences’ task (Srull & Wyer, 1979). Participants were presented with 15 strings of five words. For each word string, they had to form a sentence using four of the five words. In the materialism prime group, the word strings included words related to materialistic topics (e.g. “money”, “fashionable”, “possession”, “image”, “purchase”). In the control group, the word strings contained words relating to neutral topics (e.g., “spoke”, “sky, “chair”, “oranges”). This method has been employed by other researchers to successfully activate materialistic (Bauer et al., 2012; Zawadzka et al., 2019) and money-orientated (Vohs, 2015) mindsets. Participants were randomly assigned via Qualtrics to the materialism or control condition.

The second independent variable was whether participants were shopping for conspicuous or purely functional goods. Those in the conspicuous condition visited a hypothetical e-store called “Stylelux” which sold luxury fashion, clothing, and accessories for both men and women. The products were from designer brands (e.g., Valentino and Burberry) and many displayed visible brand logos on the item itself. The prices of items were based on those provided on the popular designer fashion e-store Net-a-porter. Those in the functional condition visited a hypothetical e-store called ‘Practical Inc’. This e-store sold products that had functional uses around the home but were not usually visible or used to display affluence, for example cleaning products, kitchen utensils, and pet food. The prices of items were based on those provided on the popular UK homeware store Wilko. Research has shown that an e-store’s layout and design, atmospherics, theatrics, and virtual social presence can all influence the extent to which consumers are likely to experience flow when using it (Hsu et al., 2013; Martins et al., 2019; Sarkar & Khare, 2019). Both websites therefore had the same layout, background colour (white) and number of items to control for factors such as interactivity, ease of use and complexity^1^.

The main dependent variables were the degree to which individuals reported experiencing (state) flow during the online shopping activity period and their subsequent attitudes and behavioural intentions towards the e-store. Measures of materialism were included before and after the priming activity. A measure of participants’ general (trait) tendency to experience flow (flow proneness) in online shopping environments was also taken so that this could be controlled for (i.e., to make sure that people are having higher quality flow experiences because they have had a materialistic mind-set activated, rather than because they are more inclined to experience flow in online shopping environments in general).

#### Measures

##### Materialistic goal orientation

Materialistic goals were measured using the 14-item shortened version of Kasser and Ryan (1996) Aspiration Index (Martos et al., 2006). This is the same scale as employed in Study 1, but participants were asked to respond with how important each goal was to them “right now”. Respondents rated how important each goal was to them on a scale of 1 (*not at all*) to 7 (*very*). The internal reliability of both the extrinsic (α = .80–.86) and intrinsic (α = .87–.89) items was good across both time points.

##### Flow Proneness

Individuals’ general tendency to experience flow within online shopping environments was measured using the Short Dispositional Flow Scale 2 (S-DFS2; Jackson et al., 2008). This is the same 9-item scale as used to measure flow proneness in Study 1. Participants were asked to rate how often they experienced each flow characteristic during online shopping in general on a scale of 1 (*never*) to 5 (*always*). The scale showed acceptable internal reliability in the present study, α = .74.

##### State Flow

Each individual’s experience of flow during the experimental shopping task was assessed using the Flow State Scale 2 (FSS2; Jackson & Eklund, 2002). The FSS2 is a 36-item questionnaire. It taps into the same nine flow characteristics as the S-DFS2 but has four items for each characteristic rather than one. Example items included “It feels like time goes by quickly” and “I am not worried about what others may be thinking of me.” Participants were asked to answer concerning the extent to which they experienced each characteristic in the shopping activity within the experiment, rather than their experiences when online shopping in general. As with the S-DFS2, each item was scored on a scale of 1 (*never*) to 5 (*always*). The scale showed excellent reliability in the present study, α = .94.

##### Attitudes Towards the E-store

Participants attitudes and behavioural intentions towards the e-store that they browsed were assessed using a combination of five items. These asked participants to rate on a scale from 1 (*strongly disagree*) to 5 (*strongly agree*) whether, if it were possible, they would continue use of the e-store in the future, they would make a purchase from the e-store, they would seek more information about the e-store, and they would spend more time browsing the e-store. Participants were also asked if they had a very positive attitude towards the company whose e-store they visited as part of the experiment. These items were selected to represent the different positive commercial consequences of flow as highlighted in existing research.

#### Procedure

Each participant completed the experiment online in a location of their choosing. The experiment was run using Qualtrics software. The experiment was introduced as a study looking to understand how people’s cognitive processes were related to their experience in online shopping environments. Participants first completed the demographic questions, S-DFS2 (measure of dispositional flow), and Aspiration Index (measure of materialistic goals). The order of these initial measures was randomised via Qualtrics. Next came the scrambled sentences task. Participants were told that on the following page they were going to be presented with 15 strings of five words. Their task was to create a sentence for each word string using four of the five words listed. An example was provided before participants began this task (e.g., the string “light sky the grey is” could be used the create the sentence “the sky is grey”). The word strings either contained the materialistic or neutral words depending upon the condition participants had been allocated to. Following completion of the scrambled sentences task, participants completed the Aspiration Index for a second time (to assess whether the priming had been effective in altering the salience of materialistic goals).

After this priming task, the activity period began. Prior to starting their activity, participants were told that their next task was to browse the online store and select at least one product for themselves and at least one product as a present for a friend or family member. Research has shown that shoppers are more likely to experience flow when they have a specific task, rather than just browsing for fun (Rettie, 2001). We asked participants to select both an item for themselves and a gift because shopping for others has been suggested to provide a higher level of challenge (Wang & Hsiao, 2012). For those in the conspicuous consumption condition, they visited the hypothetical ‘Stylelux’ e-store and were given a budget of £2000. This price was chosen to reflect the prices of items on the e-store (i.e., it meant that they could not afford all combinations of items and would have to do some budgeting). For those in the functional consumption condition, they visited the hypothetical ‘Practical Inc’ e-store and were given a budget of £40, again to reflect the prices of the items on the e-store. The instructions concerning how many products to buy, for whom, and within which budget were given to provide participants with a clear goal (one of the proposed facilitating conditions of flow). All participants spent 7 minutes on the simulated online store. The final part of the experiment involved completing the FSS2 (measure of state flow) along with the questions assessing their attitudes towards the e-store. The whole experiment took approximately 25 minutes to complete.

#### Analysis Plan

Two independent t-tests assessed differences in materialism scores both before and following the priming activity across the experimental and control groups. All hypotheses were then tested using structural equation modelling (SEM) with maximum likelihood using AMOS 23.0 (Arbuckle, 2014). Constructs that could not be directly observed (flow proneness, state flow, and attitudes/behavioural intentions towards the e-store) were treated as latent variables. In line with Matsunaga’s (2008) recommendations, flow proneness and state flow were indicated by three item parcels, which were created using the factorial algorithm method (Rogers & Schmitt, 2004). As only five items were used to assess attitudes/behavioural intentions towards the e-store, the latent variable was indicated by the five individual survey items; no parcelling was used.

The SEM was conducted in two stages. Firstly, a confirmatory factor analysis was performed on the three latent variables. This tested the validity of the measurement model, i.e., how well the latent factors were measured by the indicators. The second stage involved entering the latent variables into various structural models to assess the specific hypotheses. In all cases, models were evaluated against a combination of three established goodness-of-fit indices: the comparative fit index (CFI), the Tucker-Lewis index (TLI), and the root mean square error approximation (RMSEA). CFI and TLI values of above (or equal to) .95 and RMSEA values below .08 were considered as indicative of a good model fit (Hooper et al., 2008).

### Results and Discussion

#### Manipulation Check

Prior to the priming manipulation, the control (M = −1.96, SD = 1.20) and experimental (M = −1.74, SD = 0.98) groups did not differ in their materialism scores, *t*(138) = −1.16, *p >* .05. Following the priming manipulation, those individuals in the experimental group (M = −1.59, SD = 1.52) scored significantly higher on the materialism scale than those in the control group (M = −2.31, SD = 1.41), *t*(138) = −2.95, *p* < .01. The prime therefore appears to have been effective in enhancing the salience of materialistic values for those in the materialism group.

#### Structural Equation Modelling

Prior to specifying the measurement model, the normal distribution of the data was examined using the skewness and kurtosis coefficients. All items tested were within the acceptable limit of ±2 (Gravetter & Wallnau, 2013).

##### Reliability and Confirmatory Factor Analysis of the Measurement Model

The measurement model displayed good fit, χ2 (41) = 56.48, *p >* .05, CFI = .98, TLI = 0.98, RMSEA = 0.05. Convergent validity, which is considered to be confirmed when the Average Variance Extracted (AVE) for a construct was larger than .5, was good for all constructs. Discriminant validity, as confirmed by AVE values greater than the Maximum Shared Variance (MSV) for each construct (Hair et al., 2010), was also good for all constructs.

##### H2: The Effect of Priming Materialistic Goals on Flow Experiences

The first structural model included only the direct effect of the materialism prime condition on state flow scores, whilst controlling for the effects of age, gender, and flow proneness. This revealed a negative effect of materialistic values on flow proneness, β = −.19, *p* < .05. The model fit was good, χ^2^ (20) = 36.42, *p* < .05, CFI = .97, TLI = 0.94 and RMSEA = 0.08. This model explained 22% of the variance in state flow scores. Thus, Hypothesis 2 is not supported.

##### H3: The Moderating Effect of Shopping for Luxury versus Functional Goods

To assess the moderation effect, we started by testing for configural and metric invariance of the measurement model. To assess configural invariance, an unconstrained multi-group measurement model which allows factor loadings to vary across the functional and luxury e-store groups was created. Model fit was good, χ^2^(16) = 19.27, *p >* .05, CFI = 0.99, TLI = 0.99 and RMSEA = 0.04, implying that configural invariance was met. To assess metric invariance, a measurement model that constrained the measurement weights for each latent variable to be equal for the groups was estimated and compared to the unconstrained model. The constrained model also demonstrated good fit, χ^2^(22) = 22.42, *p >* .05, CFI = 1.00, TLI = 1.00 and RMSEA = .01. A chi-square difference test revealed that the two models were invariant, χ^2^(6) = 3.15, *p >* .05, thus supporting metric invariance.

Next, a multi-group analysis was performed to examine whether there were significant differences in the effect of materialism priming condition on state flow scores between the functional and luxury e-store groups. We compared one model, which allowed the structural paths to vary across e-store groups, with a second model, which constrained the structural path from materialism prime condition to state flow scores to be equal across e-store groups. A chi-square difference test revealed that there was not a significant difference in the size of the structural path from materialism priming condition to state flow scores across e-store types, χ^2^(1) = .21, *p >* .05.^2^ Accordingly, Hypothesis 3 is not supported.

##### H4: The Effect of Flow on E-store Attitudes and Purchase Intentions

**Figure 1.**
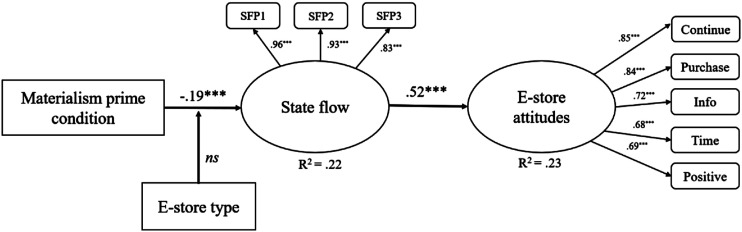
Final structural model showing that priming a materialistic mindset is negatively related to flow experiences during the shopping activity, and that flow experiences during the shopping activity are positively related to e-store attitudes. *Note.* Age, gender, and flow proneness controlled for. Error terms not displayed for the sake of clarity. All parameter values represent standardized estimates. The model accounts for 22% of variation in state flow scores and 23% of variation in e-store attitudes. *p < 0.05 **p < 0.01 ***p < 0.001. *ns* = no significant effect, *p* > 0.05. SFP1, SFP2 and SFP3 refer to State Flow Parcel 1, 2 and 3, respectively. Continue = If I could, I would like to continue use of the online store in the future. Purchase = If I could, I would make a purchase from the online store in the future. Info = If I could, I would seek out further information about the online store in the future. Time = If I could, I would have liked to spend more time browsing the online store. Positive = I have a very positive attitude towards the company whose online store I visited as part of the experiment.

To test the effect of state flow scores on attitudes and behavioural intentions towards the e-store used, the store attitudes latent variable was added into the structural model used to test Hypothesis 2 (see Figure 1). A structural path was added from the state flow score latent variable to the store attitudes latent variable, and flow proneness, age and gender were controlled for as before. This model showed good fit, χ^2^(66) = 102.30, *p* < .01, CFI = 0.96, TLI = 0.95 and RMSEA = 0.06. There was a positive effect of state flow scores on attitudes towards the e-store, β = 0.52, *p* < .01. This model explained 23% of the variance in attitudes towards the e-store scores. Hypothesis 4 is therefore supported.

Study 2 has demonstrated that increasing the salience of materialistic goals led participants to report poorer quality flow experiences in the shopping task (H2 not supported). This effect was not moderated by the type of e-store (displaying luxury/conspicuous or functional goods) used (H3 not supported). Further, in line with previous research, we found that those individuals who reported experiencing higher quality flow during the online shopping task went on to report more positive attitudes and behavioural intentions towards the e-store used (H4 supported). This research therefore demonstrates that although flow experiences during shopping can lead to desirable commercial outcomes, the materialistic goals inherent in consumer societies present a significant impediment in encouraging flow experiences in these contexts.

## Overall Discussion

This research sought to examine the relationship between materialistic goal orientation and flow experiences when shopping. Our aim was to determine whether findings showing a negative impact of materialistic goal orientation on flow experiences in more ecologically sustainable activities from the field of Environmental Psychology, would replicate in the shopping context. Several key findings have emerged from the work which have both theoretical and practical significance, which we now discuss.

There are two key empirical findings from this research. Firstly, it appears that materialistic goals can limit the extent to which people experience flow in shopping activities, both at the trait and state level. This effect appears to be present whether the shopping activity involves browsing conspicuous or functional items. Accordingly, the negative impact of materialistic goals on flow experiences seems to extend beyond those categories such as ‘work’ or ‘leisure’, or specific activities such as artwork and meditation, explored in previous research (Isham et al., 2021a, 2021b; Isham & Jackson, 2022; Isham et al., 2022), to categories such as shopping that are expected to be consistent with materialistic goals. We believe that this is the first empirical study to document this relationship within a shopping context. Secondly, the experience of flow when shopping can lead to more positive store attitudes and purchase intentions. In the present research, we replicate these effects previously documented within the field of Consumer Psychology (Ettis, 2017; Gao & Bai, 2014; Skadberg & Kimmel, 2004) using a more comprehensive measure of state flow that covers all proposed components of the flow experience.

These two findings have several ramifications. Firstly, finding that materialism still undermines flow experiences even in an activity which is consistent with materialistic goal orientations has important implications concerning the processes through which materialism undermines flow. Our findings challenge the assumption that materialism impedes flow in more sustainable activities simply because highly materialistic individuals have less interest in, liking for, or goal alignment with these activities. Instead, our findings offer support for the proposal that materialistic goals undermine flow experiences due to factors that are consistent across activity types, such as trait levels of self-regulatory strength (Isham et al., 2021b). For wellbeing practitioners, this research further highlights the importance of reducing materialistic goals to encourage beneficial flow experiences. Various strategies have been proposed to reduce the strength of materialistic goals (Kasser, 2016). These include encouraging discussion of advertising and consumption issues (Buijzen & Valkenburg, 2003), reflecting on intrinsic goals (Lekes et al., 2012), and practising gratitude (Froh et al., 2011) which enables individuals to feel more secure and thus, less susceptible to seeking comfort through material goods.

Secondly, our findings present a challenge to consumer psychologists and marketers. Our findings support the notion that the experience of flow when shopping can lead to positive commercial outcomes. However, we also document that encouraging materialistic goals, which many retail environments and advertisements often do, can undermine the quality of flow experienced when shopping. In this way, retail environments have the potential to undermine the very commercial outcomes they seek to create, by limiting opportunities for flow. For marketers, this research therefore suggests that shopping environments need to be carefully designed to encourage flow experiences. Advertisements that emphasize materialistic aspirations should be limited within online or physical stores as these could discourage flow. In line with the proposals for how to reduce the salience of materialistic goals outlined in the previous paragraph (Froh et al., 2011; Lekes et al., 2012), advertisements which emphasize ideals of community, relationships, or personal growth (rather than status, fame, and image) may be well equipped to limit the extent to which materialistic goals are salient during the shopping experience.

Just as the findings present a complex picture for marketers, they also suggest that sustainability practitioners need to be cautious before they implement broad strategies to reduce materialistic goals to promote flow. Previous work (Isham et al., 2021a; Isham & Jackson, 2022) has concluded that reducing materialistic goals may be an appropriate strategy for supporting sustainability. The present study, however, suggests that reducing materialistic goal orientation in shopping environments may lead to greater environmental impacts if it causes higher quality flow experiences when shopping and thus greater purchase intentions and behaviours. It may be better, from a sustainability perspective, to focus on reducing materialistic cues in specific contexts only (i.e., those that are more sustainable). For example, materialistic advertisements at sports grounds or in community halls should be avoided, as these contexts frequently provide opportunities for engagement in activities such as sports and crafts, which can be more sustainable sources of flow (Isham et al., 2019). This will help to encourage rewarding, flow experiences in those activities that we wish to promote engagement (i.e., those with low environmental costs) rather than in consumption-based activities.

### Limitations and Future Research

Alongside these new insights, the present research also highlights further questions to be explored in future studies addressing some of the limitations of this work. For example, one challenge to our conclusion that goal misalignment does not appear to account for why materialistic goals undermine flow could be that the shopping activity employed in the present study was not an activity deemed as important by more materialistic individuals. In Study 2, we used hypothetical online stores. This had the benefit of allowing for control over variables such as e-store layout and number of items, meaning that we could be more confident that any differences in flow experiences across the two groups were caused by the materialism prime. However, it also means that the experiment lacked ecological validity. Participants could not spend any real money and hence acquire any real products. This lack of ability to purchase real items may have limited the extent to which the shopping activity in Study 2 was aligned with materialistic goals. Future work examining real purchase decisions would help to address this criticism and further clarify the importance of goal alignment for the experience of flow. Additionally, in Study 2 participants completed the experiment online and in a location of their choosing. Whilst this had the strength of allowing the shopping activity to occur in a place more reminiscent of where participant’s real online shopping occurs, it meant we could not rule out any effects of different environments on people’s flow experiences. Future studies that run the experiment under laboratory conditions would therefore compliment the existing findings nicely.

Further, whilst Study 1 included a fairly representative sample, the experiment in Study 2 relied on a student sample which was predominantly female. The results from this specific study may therefore not be generalisable to people from a wider range of age groups and socioeconomic backgrounds. Isham et al. (2021a) demonstrated that the negative effect of priming materialistic values on subsequent flow experiences could be generalised from a student sample to a wider UK adult sample, which increases our confidence that our findings in Study 2 may also replicate across adult samples. However, there are still other demographic factors that need to be considered and explored in future research. For example, research has shown that enjoyment when shopping is more strongly associated with recommending products and brands through word-of-mouth for highly educated (vs. less educated) consumers (Mihić & Kursan Milaković, 2017). As our participants were all in the process of acquiring a university degree, we can consider them to be highly educated and, as such, it may be that experiencing the characteristics of flow when shopping may not translate into positive commercial outcomes for consumers with a lower educational background. Similarly, whilst we controlled for gender in our structural models, there was still a predominance of females in our Study 2 sample. Men and women have been reported to display different types of shopping behaviours. For example, women tend to have a more positive attitude towards leisurely browsing products whilst men perceive shopping as more of a job to be done (Tarka et al., 2022). Such differences may have impacted how they interacted with the online shopping task. Further experimental research using more diverse populations is therefore needed.

### Conclusion

This research provided the first assessment of the complex relationship between materialistic goal orientation and flow experiences in a shopping context. By documenting that materialistic goal orientation undermines flow even in the goal-congruent activity of shopping, our findings challenge the assumption that materialism impedes flow due to a lack of interest in, liking for, or goal alignment with flow-conducive activities. Rather, materialistic goals appear likely to undermine flow experiences due to factors that are consistent across activity types. Further, our results confirm previous evidence that flow during shopping can lead to enhanced commercial outcomes such as liking for a store and intentions to purchase. The combination of these two findings means that marketers need to be careful not to promote materialistic goals within shopping contexts if they are to encourage flow. Likewise, sustainability practitioners need to consider the specific contexts in which they try to reduce materialistic goals if they are to promote sustainable outcomes.

## Supplemental Material

Supplemental material - Flow Experiences in Shopping Activities: Testing Materialistic Goal Orientation as an Antecedent Supplemental material for Flow Experiences in Shopping Activities: Testing Materialistic Goal Orientation as an Antecedent by Amy Isham and Tim Jackson in Psychological Reports

## Notes

1. Visualisations from both hypothetical e-stores used in the experiment are available through the Open Science Framework: osf.io/2nw7h

2. To increase confidence that our results were not being impacted by the amount of money each participant hypothetically spent, we correlated each participant’s total spend with their dispositional flow, state flow, priming condition, and store attitudes/behavioural intentions. There were no significant correlations between money spent and any of the other variables.
