# Supplementary material for: Flow Experiences in Shopping Activities: Testing Materialistic Goal Orientation as an Antecedent
Source: Psychol Rep. 2023 Feb 23;128(2):852–80. doi: 10.1177/00332941231159615 (PMC11894835; doi:10.1177/00332941231159615)
Supplement: Supplemental material - Flow Experiences in Shopping Activities: Testing Materialistic Goal Orientation as an Antecedent [file sj-pdf-1-prx-10.1177_00332941231159615.pdf]

Flow Experiences in Shopping Activities: Testing Materialistic Goal Orientation as an Antecedent

Supplementary Materials

Supplementary Materials 1: Correlations between variables included in the regression model in Study 1

Table S1

	Age	Socioeconomic status	Education	Materialistic goals
Socioeconomic status	-.34***			
Education	.03	-.37***		
Materialistic goals	-.31***	.08*	-.10**	
Flow proneness in shopping	.09**	-.09**	.05	-.19***

Correlations Between Demographic Controls, Materialistic Goals and Flow Proneness in Shopping in Study 1

Note. * $p < .05$ ** $p < .01$ *** $p < .001$

Independent samples t-tests revealed that males (M = -1.60, SD = 1.32) tended to have slightly stronger materialistic goals than females (M = -1.81, SD = 1.47), $t(799.43) = 2.21$, $p < .05$. Further, females (M = 33.37, SD = 6.09) tended to report being more prone to experiencing flow when shopping than males (M = 32.49, SD = 6.23), $t(884) = -2.12$, $p < .05$.

Employed respondents (M = -1.50, SD = 1.44) tended to report stronger materialistic goals than unemployed respondents (M = -1.98, SD = 1.28), $t(815.09) = -5.23$), $p < .01$. There were no significant differences in the degree to which employed (M = 33.04, SD = 6.24) and unemployed (32.69, SD = 5.99), $t(873) = -.81$, $p > .05$.
